# Comparing pulsed field electroporation and radiofrequency ablation for the treatment of paroxysmal atrial fibrillation: design and rationale of the BEAT PAROX-AF randomized clinical trial

**DOI:** 10.1093/europace/euae103

**Published:** 2024-04-22

**Authors:** Nico Erhard, Eric Frison, Julien Asselineau, Besma Aouar, Serge Boveda, Hubert Cochet, Isabel Deisenhofer, Thomas Deneke, Anne Gimbert, Josef Kautzner, Sebastien Knecht, Philippe Maury, Petr Neuzil, Marine Rousset, Daniel Scherr, Christopher W Schneider, Maxime Sermesant, Dan Wichterle, Pierre Jaïs, BEAT-AF Study group, John Allison, Besma Aouar, Tiziri Aoudjit, Julien Asselineau, Laura Benkert, Serge Boveda, Christian Enzinger, Hubert Cochet, Isabel Deisenhofer, Thomas Deneke, Eric Frison, Anne Gimbert, Pierre Jaïs, Josef Kautzner, Sebastien Knecht, Michelle Lycke, Philippe Maury, Rozenn Mingam, Petr Neuzil, Maider Piquet, Sophie Regueme, Stephanie Roseng, Marine Rousset, Daniel Scherr, Christopher Schneider, Christine Schwimmer, Maxime Sermesant, Cedrick Wallet, Dan Wichterle, Besma Aouar, Tiziri Aoudjit, Julien Asselineau, Eric Frison, Thomas Gil De Muro, Anne Gimbert, Pierre Jaïs, Maria Merched, Laura Richert, Marine Rousset, Christine Schwimmer, Cédrick Wallet, Pierre Jais, Daniel Scherr, Sebastien Knecht, Petr Neuzil, Thomas Deneke, Anne Gimbert, Marine Rousset, Eric Frison, Julien Asselineau, Hubert Cochet, Dan Wichterle, Trudie Lobban, John Morgan, Besma Aouar, Thomas Gil De Muro, Maxime Sermesant, Laura Richert, Christine Schwimmer, Cédrick Wallet, Andréa Alexander, Christiane Andriamandroso, Claire Duflos, Arnaud Denis, Benoît Guy-Moyat, Nicolas Derval, Frederic Sacher, Benjamin Bouyer, Ghassen Cheniti, Meleze HOCINI, Pierre Jaïs, Philippe Maury, Anne Rollin, Serge Boveda, Stephane Combes, Jean-Paul Albenque, Josef Kautzner, Jana Haskova, Petr Peichl, Predrag Stojadinovic, Dan Wichterle, Petr Neuzil, Pavel Hala, Jan PETRU, Thomas Deneke, Elena Ene, Karin Nentwich, Isabel Deisenhofer, Felix Bourier, Florian Englert, Nico Erhard, Monika Hofmann, Marc Kottmaier, Sarah Lengauer, Tilko Reents, Jan Syvari, Marta Telishevska, Alex Tunsch Martinez, Daniel Scherr, Martin Benedikt, Anna-Sophie Eberl, Martin Manninger-Wuenscher, Ursula Rohrer, Sebastien Knecht, Mattias Duytschaever, Jean-Benoît Le Polain de Waroux, René Tavernier, Thomas Deneke, Daniel Scherr, Christian Enzinger, Dan Wichterle, Sébastien Knecht, Hubert Cochet, Maxime Sermesant, Vigneshwar Gurunathan, Julien Castelneau

**Affiliations:** Department of Electrophysiology, German Heart Center Munich, Lazarettstraße 36, 80636 Munich, Technical University of Munich, Munich, Germany; University Bordeaux, INSERM, Institut Bergonié, CHU Bordeaux, CIC 1401, EUCLID/F-CRIN Clinical Trials Platform, Bordeaux, France; University Bordeaux, INSERM, Institut Bergonié, CHU Bordeaux, CIC 1401, EUCLID/F-CRIN Clinical Trials Platform, Bordeaux, France; University Bordeaux, INSERM, Institut Bergonié, CHU Bordeaux, CIC 1401, EUCLID/F-CRIN Clinical Trials Platform, Bordeaux, France; Heart Rhythm Department, Clinique Pasteur, Toulouse, France; IHU LIRYC, University Bordeaux, CHU Bordeaux, Bordeaux, France; Department of Electrophysiology, German Heart Center Munich, Lazarettstraße 36, 80636 Munich, Technical University of Munich, Munich, Germany; Department of Cardiology, Cardiovascular Center Bad Neustadt/Saale, Bad Neustadt an der Saale, Germany; Clinical Research and Innovation Department, CHU Bordeaux, Bordeaux, France; Department of Cardiology, Institute for Clinical and Experimental Medicine, Prague, Czechia; Department of Cardiology, AZ Sint-Jan Hospital, Bruges, Belgium; Department of Cardiology, University Hospital Rangueil, Toulouse, France; Cardiology Department, Na Homolce Hospital, Homolka Hospital, Prague, Czechia; Clinical Research and Innovation Department, CHU Bordeaux, Bordeaux, France; Division of Cardiology, Medical University of Graz, Graz, Austria; Department of Electrophysiology, Boston Scientific Corporation, St Paul, MN, USA; IHU LIRYC, University Bordeaux, INSERM, CRCTB, U 1045, Bordeaux, France; Inria, Université Côte d'Azur, Epione Team, Sophia Antipolis, France; Department of Cardiology, Institute for Clinical and Experimental Medicine, Prague, Czechia; IHU LIRYC, University Bordeaux, CHU Bordeaux, Bordeaux, France

**Keywords:** Atrial fibrillation, Catheter ablation, Pulmonary vein isolation, Pulsed field ablation, Radiofrequency current, Randomized trial

## Abstract

**Aims:**

Using thermal-based energy sources [radiofrequency (RF) energy/cryo energy] for catheter ablation is considered effective and safe when performing pulmonary vein isolation (PVI) in patients with paroxysmal atrial fibrillation (AF). However, treatment success remains limited and complications can occur due to the propagation of thermal energy into non-target tissues. We aim to compare pulsed field ablation (PFA) with RF ablation in terms of efficacy and safety for patients with drug-resistant paroxysmal AF.

**Methods and results:**

The BEAT PAROX-AF trial is a European multicentre, superiority, open-label randomized clinical trial in two parallel groups. A total of 292 participants were recruited in 9 high-volume European clinical centres in 5 countries. Patients with paroxysmal AF were randomized to PFA (FARAPULSE Endocardial Ablation System©, Boston Scientific) or RF using the CLOSE protocol with contact force sensing catheter (SmartTouch© catheter and CARTO© Biosense Webster). The primary endpoint will be the 1-year recurrence of atrial arrhythmia, and the major secondary safety endpoint will be the occurrence of acute (<7 days) procedure-related serious adverse events, or pulmonary vein stenosis, or atrio-oesophageal fistula up to 12 months. Additionally, five sub-studies investigate the effect of PFA on oesophageal safety, cerebral lesions, cardiac autonomic nervous system, durability of PVI as assessed during redo ablation procedures, and atrial and ventricular function. The study began on 27 December 2021 and concluded recruitment on 17 January 2024. Results will be available in mid-2025.

**Conclusion:**

The BEAT PAROX-AF trial aims to provide critical insights into the optimal treatment approach for patients with paroxysmal AF.

## Graphical Abstract

**Graphical Abstract:**
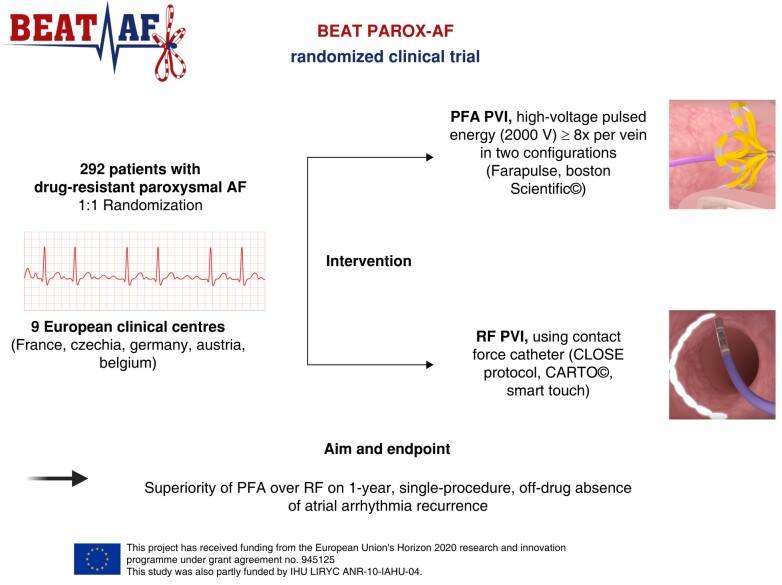
Created with BioRender.com

## Introduction

Performing catheter ablation has emerged as a well-excepted therapeutic approach for the treatment of patients with symptomatic paroxysmal atrial fibrillation (AF).^1–3^ Thermal-based energy sources [radiofrequency (RF) energy/cryo energy] for catheter ablation are considered effective and reasonably safe in this indication.^2,4^ However, treatment success remains limited with single-procedure success rates between 60% and 80%.^5–8^ Pulmonary vein reconnection is believed to be a main underlying factor limiting treatment success. Therefore, creating more durable lesions when performing catheter ablation has been a major focus of scientific investigation within the field of clinical electrophysiology over the last decade.^9,10^ Furthermore, thermal ablation strategies can result in rare yet significant complications since these methods ablate all types of tissue in a non-selective manner.^11–13^ Pulsed field ablation (PFA) is a novel, non-thermal ablation strategy based on pulsed electric field (PEF) energy delivery. This energy source allows highly selective cardiac ablation that resulted in safe, durable, and transmural lesions as demonstrated in smaller studies.^14,15^ The BEAT PAROX-AF trial aims to provide comparative evidence of PEF vs. RF ablation in terms of efficacy and safety when treating patients with paroxysmal AF. The primary objective of the trial is to test the superiority of PFA over standard RF catheter ablation on the rate of 1-year recurrence of atrial arrhythmia. We hypothesize that PEF energy, a non-thermal ablative modality highly specific for cardiac cells, will deliver more durable PVI than RF energy, translating into superior clinical outcomes for PFA as compared with RF energy. By evaluating the outcomes of PFA, the study aims to provide critical insights into the optimal treatment approach for paroxysmal AF, to improve their clinical outcomes, including quality of life, and procedural duration.

## Methods

### Study design, setting, and recruitment

The BEAT PAROX-AF trial is a European multicentre, prospective, superiority, open-label randomized clinical trial with two parallel groups (PEF vs. RF catheter ablation). The study protocol is registered on clinicaltrials.gov (NCT05159492) and has been approved by the local ethics committee for all participating centres. The present report adheres to the SPIRIT 2013 Statement.^16^ Participants were recruited in nine high-volume European clinical centres in five countries (France, Czechia, Germany, Austria, and Belgium*)* (see Supplementary material for details on all participating centres). All centres have extensive experience in performing both types of ablation strategies. To attenuate the learning effect in PVI using PFA, participating centres started enrolling in the BEAT PAROX-AF trial after at least three PFA with study devices, either in clinical routine practice or during the study-specific training period. Six of the centres perform ∼600 AF ablations annually, while the rest do over 300 cases per year.

### Study population

Patients aged ≥18 and ≤80 years with drug-resistant and documented symptomatic paroxysmal AF, indication for AF ablation, and effective oral anticoagulation >3 weeks before the planned ablation procedure were eligible for inclusion. Main exclusion criteria were persistent AF, paroxysmal AF secondary to electrolyte imbalance, thyroid disease, alcohol or other reversible/non-cardiac causes, left atrial (LA) anteroposterior diameter ≥ 5.5 cm, or any prior atrial endocardial or epicardial ablation procedure except for the ablation of cavotricuspid isthmus (CTI)-dependent flutter or right-sided supraventricular tachycardia, PV abnormality, stenosis or stenting, and refusal to give informed consent. Detailed eligibility criteria are listed in the Supplementary material.

### Interventions

In both groups, patients were under conscious sedation (benzodiazepine, morphine), and boluses of propofol were injected just before energy deliveries. General anaesthesia, including intubation, was not used. Procedures will include transseptal puncture in the absence of foramen ovale.

### Pulmonary vein isolation using pulsed field ablation

**Figure 1:**
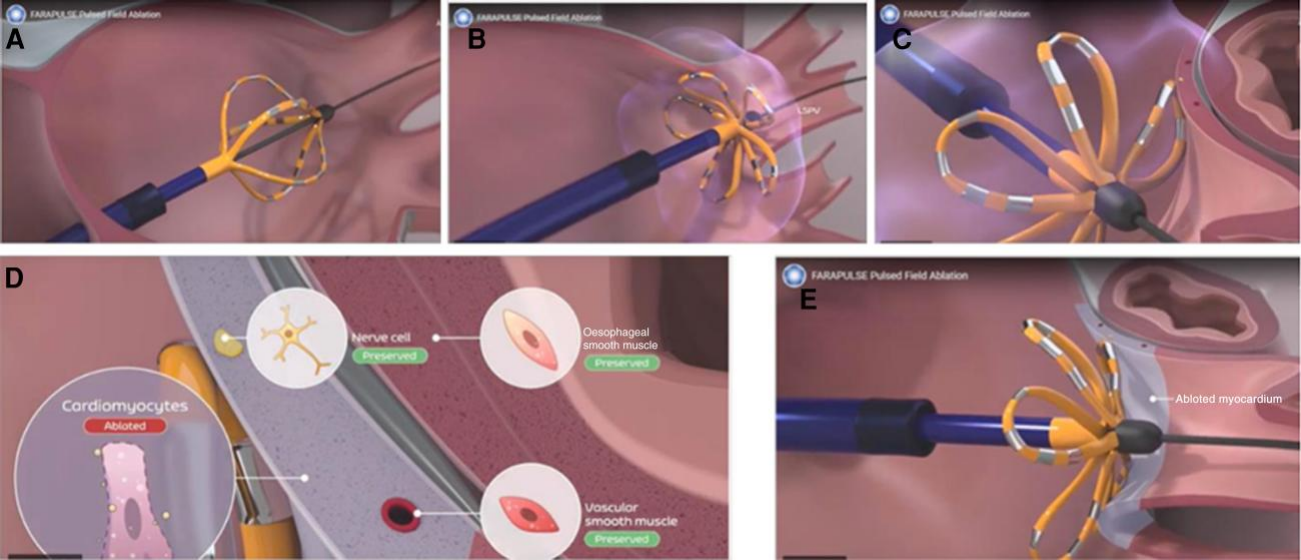
Performing pulmonary vein isolation with the FARAPULSE© PFA system.

Through the precise and momentary creation of a therapeutic electric field, PFA treats the uniquely sensitive myocardium (*Figure 1D*). Collateral, non-target tissues are spared due to their higher threshold to PEF.^15,17^ The FARAPULSE Endocardial Ablation System (Boston Scientific©) includes the FARAWAVE Endocardial Ablation Catheter System, the FARASTAR Endocardial Generator System, and the FARADRIVE Deflectable Sheath System (*Figure 1*). The 12 F over-the-wire PFA catheter has five splines that each contain four electrodes and can be deployed in either a basket (*Figure 1A*) or a flower configuration (*Figure 1B*). The catheter is advanced over a guidewire such that the splines achieve circumferential contact/proximity with the PV antra (*Figure* *1B* and *C*). The ablative energy is delivered from all electrodes. The catheter is rotated between applications to ensure circumferential PV ostial and antral coverage. Energy (2000 V) was delivered at least eight times per vein with two different catheter configurations and rotations (flower and basket configuration). Before starting ablation, activated clotting time is aimed to be above 300 s. After performing a transseptal puncture, all four PVs were isolated, demonstrated by an entrance and/or exit block. If subjects have a history of typical flutter or have an inducible typical flutter, they underwent CTI interruption using RF. Ablation of an accessory pathway, atrioventricular nodal re-entrant tachycardia, treatment of incidental left-sided atrial flutter, or incessant atrial tachycardia may be performed at the investigator’s discretion if required for subject welfare, using RF and not PEF. During the index procedure for paroxysmal AF, no other lesions were attempted.

### Pulmonary vein isolation using contact force radiofrequency

The utilization of RF energy to perform PVI is a widely accepted treatment option and continues to be the current ‘gold standard’ when performing catheter ablation procedures. To do so, the CARTO© (Biosense Webster) platform was used along with a contact force catheter (SmartTouch or SmartTouch Surround Flow), to achieve an ablation index value of 300–400 and at least 500 at the posterior and anterior segments of PV antra, respectively. Power was restricted to 35/45 W with a distance of 6 mm or less between consecutive RF deliveries in accordance with the CLOSE protocol.^18^ Pulmonary vein isolation demonstrated by the entrance and/or exit block was the endpoint of the procedure.

### Redo procedure

If redo procedures are needed, they will be performed using RF in both groups as described above. Pulmonary vein reconnections, lesion set, and arrhythmia mechanism (if applicable) will be documented. This will potentially require the alcoholization of the vein of Marshall and the creation of a mitral isthmus line or any other clinically relevant ablation strategy.

## Endpoints

### Primary endpoint

The primary efficacy endpoint was defined as the proportion of subjects experiencing 1-year single-procedure clinical success, defined as per the 2017 Heart Rhythm Society (HRS) consensus statement: successful index AF ablation, absence of atrial arrhythmia recurrence ≥30 s on any type of recording [transtelephonic monitoring (TTM), Holter recording, 12-lead electrocardiogram (ECG), rhythm strip, or other diagnostic ECG documentation], absence of use of class I or III antiarrhythmic drugs (AAD) except for non-atrial arrhythmia, and absence of redo ablation (except for typical flutter), in the 12 months following the index ablation procedure.^4^ A blanking period of 60 days for arrhythmia recurrence after the index ablation procedure will be systematically used, as is usual in AF ablation.^19^ Recurrences of atrial arrhythmia during the blanking period will not be considered in the determination of the primary endpoint. However, any redo ablation for atrial arrhythmia except for typical flutter performed during or after the blanking period will be considered a failure.

### Secondary endpoints

Secondary efficacy endpoints are as follows:

1. The proportion of subjects with 1-year multiple-procedure success defined as:
  - Absence of atrial arrhythmia recurrence ≥30 s on any type of recording as defined above
  - Absence of use of class I or III AAD (except for non-atrial arrhythmia)
2. Improvement in quality of life, using a standard set of patient-reported questionnaires for paroxysmal AF already validated in the different native languages of the participating centres^20^: general health-related quality of life (physical and mental components of the SF-12 score measured at 6 and 12 months^21^) and AF-related quality of life assessed with the Atrial Fibrillation Effect on QualiTy-of-life (AFEQT) questionnaire at 6 and 12 months.^22^ The AFEQT is composed of 20 items covering 4 individual domains: symptoms (4 items), daily activities (8 items), treatment concern (6 items), and treatment satisfaction (2 items). The AFEQT global score (using the first three domains as recommended) and scores for each of the three subdomains (symptoms, daily activities, and treatment concern) will be measured.
3. Proportion of participants with death, strokes, or embolic events from arrhythmia up to 12 months after the index ablation procedure.

The secondary safety endpoint is the proportion of participants with one or more of the following ablation procedure-related serious adverse events (SAE), as adjudicated by a blinded Clinical Event Committee:

1. Early-onset SAE (within 7 days of index ablation procedure): death, myocardial infarction, persistent diaphragmatic paralysis, stroke, transient ischaemic attack (TIA), peripheral or organ thromboembolism, cardiac tamponade or perforation, pericarditis, new hospitalization (excluding those solely due to arrhythmia recurrence), prolonged index hospitalization, heart block, or vascular access complications.
2. Late-onset SAE (at any time through 12-month follow-up): PV stenosis or atrio-oesophageal fistula.

Other secondary endpoints are the duration of the AF ablation procedure (mean total procedure duration, LA dwell time, mean fluoroscopy time) and 1-year healthcare resource utilization (all-cause hospitalizations, AF-related and non–AF-related cardiovascular hospitalizations, repeat ablations, direct current cardioversions). Among exploratory endpoints belong the change in mean PV diameter from baseline to 2 months after the index ablation and the impact of ablation on the indices of cardiac autonomic regulations.

### Study schedule

The study schedule is summarized in *Table 1*.

**Table 1:** Study schedule summary for the BEAT PAROX-AF trial and sub-studies

Assessment	Screening visit^a^	Baseline visit^a^	Index ablation procedure	Pre-discharge visit		1-Month phone call	2-Month visit	Redo procedure (if needed)	6-Month visit	12-Month visit	Unscheduled visit
	−2 months to D-1	D-1 to D0	D0	D1	D1 to D3	D30 (±7)	D60 (±15)	D60 to D365	D180 (±30)	D365 (±30)	
Eligibility criteria	X	X									
Informed consent	X	X									
CT/TEE to exclude LA thrombus		X									
Randomization		X^b^									
Procedure characteristics			PEF or RF					RF only			
AAD and anticoagulant medications	X	X		X		X	X		X	X	X
Recurrent arrhythmia, cardioversions, hospitalizations				X		X	X		X	X	X
12-lead ECG		X		X			X		X	X	X
24 h Holter							X		X	X	
Transtelephonic ECG monitoring					Weekly and in case of symptoms						
Cardiac CT/MRI		X					X				
TTE		X								X	
NYHA classification		X		X		X	X		X	X	X
Fluoroscopic examination of the diaphragm			X				X^c^		X^c^	X^c^	
QoL questionnaires		X							X	X	
Adverse events		X	X	X		X	X	X	X	X	X
Sub-study examinations											
Oesophagoscopy					X						
Brain MRI					X		X^d^				
24 h Holter		X									
Extracardiac vagal stimulation			X								
Atropine test		X		X					X	X	
CARTO export			X					X			
Cardiac MRI		X	X				X				

AAD, antiarrhythmic drug; CT, computed tomography; LA, left atrial; MRI, magnetic resonance imaging; NYHA, New York Heart Association; PEF, pulsed electric field; QoL, quality of life; RF, radiofrequency; TEE, transoesophageal echocardiography; TTE, transthoracic echocardiography.

^a^The screening visit and the baseline visit could be done the same day.

^b^Randomization may be performed up to 10 days before Day 0 if required for ablation scheduling.

^c^If the procedural examination indicates decreased phrenic nerve function.

^d^For participants with cerebral lesions on baseline brain MRI.

### Screening

Patients scheduled for AF consultation or ablation were screened in the cardiac electrophysiology departments. The documentation of eligibility criteria required the collection of data from the patient’s medical history, transthoracic echocardiography (TTE) and cardiac CT, or magnetic resonance imaging (MRI) results. Eligible patients willing to participate after reading the information sheet were asked to give written informed consent witnessed by a principal or co-investigator.

### Randomization and allocation concealment

The randomization list was established by the biostatistician of the EUCLID platform (CHU Bordeaux) before the start of the trial and kept confidential in a secure environment. Randomization was balanced (1:1 ratio) and stratified by centre. Full details of the randomization scheme were not disclosed to ensure allocation concealment and were included in a separate document with restricted access, kept by the biostatistician. Participants were included during pre-operative consultation, and eligible patients were randomized preferentially the day before or the day of the procedure or up to a maximum of 10 days in advance to facilitate ablation scheduling. Randomization was conducted through the trial electronic case report form (eCRF), after documentation of the eligibility criteria. The assigned intervention was communicated to participants on-site before the AF ablation procedure to minimize the likelihood of withdrawals between randomization and intervention.

### Blinding

Due to the nature of the intervention and important differences between PFA and RFA, the blinding of patients and clinical staff is not possible. However, a centralized assessment (brain MRI, cardiac MRI, video-recording of oesophagoscopy, Holter, TTM) is performed blinded to allocated intervention whenever possible, to limit the probability of differential evaluation between the two groups.

### Follow-up visits

Patients will be followed with a phone call at 1 month and outpatient visits at 2 months, 6 months, and 12 months. At each in-hospital visit, medication, symptoms of arrhythmia, and adverse events will be collected, a physical examination will be performed, and a 12-lead ECG, as well as a 24 h Holter monitor recording, will be obtained. Furthermore, self-performed single-lead ECGs will be conducted weekly and in case of symptoms via a smartphone application and then sent to the clinical centre for day-to-day care and to the centralized core lab for further evaluation. A fluoroscopic examination of diaphragm motion will be performed during follow-up if the procedural examination indicated phrenic nerve damage. All patients will undergo cardiac computed tomography (CT) or cardiac MRI at the 2-month follow-up to assess PV stenosis. Computed tomography protocols will consist of a conventional arterial-enhanced ECG-gated acquisition. Magnetic resonance imaging protocols will consist of a three-dimensional angiography acquisition with respiratory navigation. The diameter analysis of all PVs will be centralized by a core lab at the University of Bordeaux. SF-12 and AFEQT will be collected at the 6-month and 12-month follow-up visits, and a TTE will be performed at 12 months. A redo procedure will be collected, as well as any unscheduled visit for AF.

### Sample size

Radiofrequency has been reported to provide around 65% single-procedure success rate in paroxysmal AF.^23,24^ A success rate of 80% could be expected with PEF.^25^ With a type I error rate of 5%, a power of 80%, and randomization with a 1:1 ratio, we need to include 276 participants (138 participants in each arm). After accounting for a maximum of 5% of missing data at 12 months, the total sample size was increased to 292 participants (146 participants in each arm).

### Statistical methods

Primary analyses will be conducted according to the intention-to-treat principle, in which all randomized patients will be included in the arm in which they first were randomized, and all their data will be used. The comparisons of the single-procedure 1-year success rates between the PEF arm and the RF arm will use logistic mixed models with a random effect on the study centres and fixed effects on the procedure arms, with a two-sided type I error rate of 5%. The model will be adjusted on major prognostic factors (common ostia, intermediate PV, PV diameter > 25 mm, and body mass index). For secondary objectives, linear (for quantitative outcomes) and logistic (for binary outcomes) mixed models will be used to compare the outcomes between the arms. The assumption of the models will be systematically checked.

### Sub-studies

As of today, many data exist regarding possible risks and complications when using conventional thermal energies for AF ablation. In contrast, few data and no randomized assessments are available regarding the use of PFA. In particular, studies investigating PFA and oesophageal safety, PFA and the risk of cerebral lesions, its impact on vagal response, and the autonomic nervous system (ANS) are very limited. Additionally, we aimed to assess the need for redo procedures related to PV reconnection and recurrent arrhythmia mechanisms as well as the impact of PFA on atrial and ventricular function. Five sub-studies were thus designed for these objectives. Participants in the main study were offered optional participation in sub-studies. Informed consent is given separately for each sub-study and separately from the main study. Participants are free to take part in the main study without participating in any of the sub-studies, and they are free to participate in as many sub-studies as they wish, among those offered in their site.

### Oesophageal safety

We hypothesize that PFA is associated with fewer or no oesophageal lesions, as compared with RF. Participants were offered systematic oesophagoscopy 1–3 days after index ablation in both arms. As atrio-oesophageal fistulas are rare, oesophageal lesions related to AF ablation will be used as a surrogate, and we will compare their incidence and characteristics, between PFA and RF.^13^ The video recordings will be centrally analysed and categorized by a core lab blinded to the study procedure. In patients enrolled into ‘Atrial and ventricular imaging’ sub-study (see below), acute MRI will also be used to assess acute oesophageal injury after ablation.

### Brain magnetic resonance imaging

In this sub-study, we postulate that PFA—being a non-thermal catheter ablation modality—is not associated with an increased risk for cerebral lesions as opposed to RFA and there are no differences in the evolution of lesions. The sub-study objectives will be to compare groups on the occurrence of acute ischaemic or haemorrhagic cerebral lesions and their 2-month evolution, as assessed with brain MRI. Participants with symptomatic cerebral events (stroke/TIA) will be subjected to acute brain MRI dictated by clinical reasoning. All sub-study participating patients underwent a brain MRI (diffusion-weighted imaging, fluid attenuated inversion recovery, susceptibility-weighted imaging) on Days 1–3 following the index ablation procedure. In addition, those patients with positive baseline MRI (demonstrating acute ischaemic or haemorrhagic lesions) will have a follow-up MRI after 2 months. All MRIs will be analysed centrally and blinded to the study procedure.

### Autonomic nervous system

We expect that PFA has a less significant impact on cardiac ANS function compared with thermal energy sources. For this investigation, we aim to describe the acute and 1-year change in cardiac ANS outcomes. Participants in this sub-study underwent a limited intraprocedural EP study, including extracardiac right vagal nerve stimulation to evaluate the extent of vagal denervation (immediately after left and right PV ablation). In addition, they will undergo serial atropine testing and heart rate variability assessment up to 12 months of follow-up.

### Redo procedures

The frequency of redo procedures is expected to be less in the PFA group, mostly because of more durable lesions and thus fewer PV reconnections. We aim to evaluate the prevalence of electrical PV reconnection after PFA as compared with standard RF. We also want to understand if recurrent atrial arrhythmias are associated with PV reconnections and to determine if the recurrent arrhythmia mechanisms are different after PFA vs. RF index ablation. Therefore, high-density CARTO maps from redo procedures (as clinically indicated) will be analysed in detail.

### Atrial and ventricular imaging

We expect unchanged atrial morphology (including PV diameter) and function when PVI is performed with non-thermal PFA energy. This sub-study will compare the impact of PFA vs. RF on LA function, left ventricular (LV) structure and function, and acute and chronic tissue changes after ablation. Participants will be offered high-resolution cardiac MRI (cine and late gadolinium enhancement) at baseline and 2 months, and a sub-set of participants will be offered an additional acute cardiac MRI just after the index ablation. Cine images will be processed to assess atrial strain, reservoir, and contractile LA function as well as LV ejection fraction.

### Data management

Different tasks of data management (from study design to database closure) and the responsibilities of each person involved in the data management process and quality control are detailed in a data management plan. Clinical data are collected by using an eCRF (screening, baseline, and follow-up data). Any original document or information recorded during the study is defined as a source document, and the eCRF must accurately reflect the data in the source document. Source documents in the framework of the study can be paper CRF, original copies of scales, or medical files. Consistency checks will be programmed by the data manager to check the consistency and the completion of data in the eCRF. The list of consistency checks will be predefined by the project team and passed on to the data manager who will write a study-specific data validation plan. Additional queries might also be raised by the clinical research assistant. Queries are sent to the clinical site via the eCRF. Remote and on-site monitoring is organized throughout the trial to ensure compliance with the protocol, regulations, and good clinical practice recommendations. Complex data comprising images (MRI, CT, TTE, X-rays, endoscopy) and signals (ECG, Holter, electroanatomic mapping) will be pseudonymized by each clinical partner using participant ID and stored in the Electrophysiology and Heart Modelling Institute (Lyric) of the University of Bordeaux (UBx) (UBx-Liryc) database.

### Auditing

Audits of the trial will be periodically arranged by reviewing the data obtained as well as the procedural aspects of the trial. This may include on-site audits and source data checks. Direct access to source documents is required for these periodical inspections. An audit may be performed at any time by persons authorized by the sponsor and independent of the person in charge of the trial. This audit may be performed at the sponsor site and/or on-site and includes a review of source data. For the same purpose, an inspection may be done at any time, by representatives of the relevant health authorities.

### Current state of progress

The recruitment period was from 27 December 2021 (inclusion of the first participant) to 17 January 2024 (inclusion of the last participant). Follow-up is ongoing and expected to be completed by the end of January 2025, with communication of results by mid-2025.

### Protocol amendments

Since the first approved version of the protocol (version 1.0 of 13 September 2021), several protocol amendments were made. Major changes were related to eligibility criteria (documentation of paroxysmal AF, documentation of LA diameter and LV ejection fraction, contraception, formalization of the indication for PVI/AF ablation), study examination and randomization timeframes to resolve scheduling difficulties, precisions on the investigational procedure and redo procedures, and authorized treatments and procedures. The currently approved version is version 7.0 of 19 June 2023.

## Discussion

The BEAT PAROX-AF trial is a prospective multicentre randomized trial which aims to compare the use of PFA with standardized RF ablation (using the CLOSE protocol) in patients receiving their first-time catheter ablation for symptomatic drug-refractory AF. Over the last decades, catheter ablation to achieve PVI has emerged as the ‘cornerstone’ strategy when treating symptomatic AF.^2–4^ To achieve reliable PVI, several energy sources have been developed, all to deliver durable lesions while also proving high patient safety and an effective procedural workflow. As of today, the most commonly used ablation modalities are RF or cryo-ablation-based, using conductive heating or cooling to destroy the targeted tissue.^26,27^ The FIRE AND ICE trial from 2016 which compared these aforementioned ablation strategies has demonstrated ablation using a cryo-balloon to be non-inferior in regard to efficacy and safety compared with standard RF-based ablation in patients with paroxysmal AF.^5,28^ Single-procedure 1-year success rates of both ablation modalities are estimated to range between 50% and 80%. Patients with persistent AF have notably lower success rates compared with those with paroxysmal AF.^2,4,6^ In elite single-centre reports, the success rate for eliminating symptomatic AF (inclusive of both paroxysmal and persistent AF) varies from 55–77%, with many patients requiring multiple procedures.^24,29,30^ New ablation techniques and devices continue to be developed to improve safety and efficacy while reducing procedure time and complexity. Over recent years, PFA has emerged as a novel non-thermal energy source using electroporation to create durable ablation lesions while also providing cardioselectivity leaving other tissues unharmed.^14,15,31^ In a 2021 multicentric safety and feasibility study which enrolled 121 patients with paroxysmal AF, Reddy *et al.* reported a 1-year freedom from any atrial arrhythmia of 84.5 ± 5.4% when using optimized biphasic energy with a single-shot PFA device. Primary adverse events were low at 2.5% with procedure times averaging 92 min.^15,32^ Furthermore, results of several studies indicate that, unlike thermal-based ablation strategies, PFA does not seem to induce any form of oesophageal or nerve injury.^14,31,33,34^ The ADVENT trial which was published in late 2023 is the first prospective randomized non-inferiority trial to compare PFA with thermal ablation strategies (RF ablation or cryo-balloon ablation) in patients with paroxysmal AF.^35^ In this US-based trial, PFA was non-inferior to thermal-based ablation concerning the composite primary efficacy endpoint of initial procedural failure, documented atrial tachyarrhythmia after a 3-month blanking period, AAD use, cardioversion, or repeat ablation.^35^ Importantly, the operators in this trial had limited experience with PFA and many were still in the learning curve phase with this technology as compared with many years of practice using thermal strategies.^35^ The BEAT PAROX-AF trial aims to demonstrate PFA superiority over RF in a European setting. The operators in this trial have performed a significant number of PFA cases prior to enrolling patients into the study. Furthermore, the predefined sub-studies will further enhance our in-depth understanding of this promising new technology. Overall, PFA has the potential to revolutionize the field of cardiac electrophysiology, promising to provide a safer, more effective, and faster ablation strategy when compared with conventional ablation modalities. Therefore, the BEAT PAROX-AF trial and its sub-studies aim to provide much-needed reliable randomized data regarding PFA and critical insights into the optimal treatment approach for paroxysmal AF.

## Supplementary Material

euae103_Supplementary_Data

## Clinical trial registration number

Clinicaltrials.gov, NCT05159492.
