# Supplementary material for: Comparing pulsed field electroporation and radiofrequency ablation for the treatment of paroxysmal atrial fibrillation: design and rationale of the BEAT PAROX-AF randomized clinical trial
Source: Europace. 2024 Apr 22;26(5):euae103. doi: 10.1093/europace/euae103 (PMC11068269; doi:10.1093/europace/euae103)
Supplement: euae103_Supplementary_Data [file euae103_supplementary_data.docx]

**SUPPLEMENTARY MATERIAL**

1. **List of participating centres**

9 European clinical sites managed by 7 clinical sites (beneficiaries) from 5 related countries:

| **Clinical Centres** | **Country** | **Principal Investigator** |
| --- | --- | --- |
| Centre Hospitalier Universitaire (CHU) Bordeaux | France | Nicolas Derval |
| Centre Hospitalier Universitaire (CHU) Toulouse | France | Philippe Maury |
| Clinique Pasteur, Toulouse | France | Serge Boveda |
| Institute for Clinical and Experimental Medicine, Prague | Czechia | Josef Kautzner |
| Homolka Hospital, Prague | Czechia | Petr Neuzil |
| RHÖN-KLINIKUM Campus Bad Neustadt, Bad Neustadt | Germany | Thomas Deneke |
| Deutsches Herzzentrum München, Munich | Germany | Isabel Deisenhofer |
| Medical University of Graz, Graz | Austria | Daniel Scherr |
| AZ Sint-Jan Brugge-Oostende, Bruges | Belgium | Sebastien Knecht |

1. **Eligibility Criteria**

**Inclusion Criteria**

1. Patients with drug-resistant symptomatic paroxysmal AF meeting all the following criteria:
   1. Paroxysmal: AF that terminates spontaneously or with intervention within 7 days of onset.
   2. Frequency: At least one (1) paroxysmal AF episode documented by a recording such as ECG, Event Monitor, Holter monitor or telemetry strip. Note: A medical report from a cardiologist indicating the presence of a paroxysmal AF episode on a recording already performed, even if the recording is not provided, is accepted.
   3. The drug failed: Failed AAD treatment, meaning therapeutic failure of at least one (1) AAD (Class I to IV) for efficacy and/or intolerance.
   4. Indication for PVI/AF ablation
2. Patients who are ≥18 and ≤80 years of age on the day of enrolment.
3. Patients who are willing and capable of:
   1. Providing informed consent to undergo study procedures AND
   2. Participating in all examinations, follow-up visits and tests associated with this clinical study.
   3. Having a smartphone compatible with the Event Monitor device.
4. Highly effective contraception for women of childbearing potential, maintained during research procedures.
5. Effective oral anticoagulation >3 weeks before the planned ablation procedure
6. Patient affiliated to or beneficiary of national health security scheme for French and Belgian participants.

**Non-inclusion criteria**

1. AF that is any of the following:
   1. Persistent (both early and longstanding) by diagnosis or continuous duration >7 days
   2. Secondary to electrolyte imbalance, thyroid disease, alcohol or other reversible / non-cardiac causes
2. Any of the following atrial conditions:
   1. LA anteroposterior diameter ≥5.5 cm or LA end-systolic volume ≤10 ml/m² or ≥50ml/m² (by MRI, CT or TTE)
      Note: A medical report from a cardiologist/radiologist indicating the LA size is normal or only slightly enlarged/dilated, is accepted.
   2. Any prior atrial endocardial or epicardial ablation procedure, other than right sided cavotricuspid isthmus ablation or for right sided SVT
   3. Any prior atrial surgery
   4. Intra-atrial septal patch or interatrial shunt
   5. Atrial myxoma
   6. Current LA thrombus
   7. LA appendage closure, device or occlusion, past or anticipated
   8. Any PV abnormality, stenosis or stenting (common and middle PVs are admissible)
3. At any time, one (1) or more of the following cardiovascular procedures, implants or conditions:
   1. Sustained ventricular tachycardia or any ventricular fibrillation
   2. Hemodynamically significant valvular disease:
      1. Valvular disease that is symptomatic
      2. Valvular disease causing or exacerbating congestive heart failure
      3. Aortic stenosis: if already characterized, valve area <1.5 cm or gradient >20 mm Hg
      4. Mitral stenosis: if already characterized, valve area <1.5 cm or gradient >5 mm Hg
      5. Aortic or mitral regurgitation associated with abnormal LV function or hemodynamic measurements
   3. Hypertrophic cardiomyopathy, LV wall >15 mm
   4. Any prosthetic heart valve, ring or repair including balloon aortic valvuloplasty
   5. Pacemaker, implantable cardioverter defibrillator or cardiac resynchronization therapy devices
   6. Inferior vena cava filter or known inability to obtain vascular access, or other contraindication to femoral access
   7. History of rheumatic fever
   8. History of congenital heart disease with any residual anatomic or conduction abnormality
4. Any of the following procedures, implants or conditions:
   1. At baseline:
      1. New York Heart Association (NYHA) Class III/IV
      2. LV ejection fraction <40%
         Note: A medical report from a cardiologist/radiologist indicating the LV ejection fraction is normal, preserved or only slightly altered/reduced, is accepted.
      3. Symptomatic hypotension
      4. Uncontrolled hypertension (systolic blood pressure [BP] >160 mmHg or diastolic BP >95 mmHg on two BP measurements at baseline assessment)
      5. Symptomatic resting bradycardia
      6. Implantable loop recorder or insertable cardiac monitor,
   2. Within the 3 months preceding the Consent Date:
      1. Myocardial infarction
      2. Unstable angina
      3. Percutaneous coronary intervention
      4. Heart failure hospitalization
      5. Pericarditis or symptomatic pericardial effusion
      6. Gastrointestinal bleeding
   3. Within the 6 months preceding the Consent Date:
      1. Heart surgery
      2. Stroke, TIA or intracranial bleeding
      3. Any thromboembolic event
      4. Carotid stenting or endarterectomy
5. Diagnosed disorder of blood clotting or bleeding diathesis
6. Contraindication to, or unwillingness to use, systemic anticoagulation
7. Contraindications to both CT and MRI
8. Sensitivity to contrast media not controllable by premedication
9. Women who are pregnant, lactating, or who are planning to become pregnant during the anticipated study period
10. Medical conditions that would prevent participation in the study, interfere with assessment or therapy, significantly raise the risk of study participation, or modify outcome data or its interpretation, including but not limited to:
    1. Body mass index >40.0 kg/m^2^
    2. Solid organ or hematologic transplant, or currently being evaluated for an organ transplant
    3. Severe lung disease, pulmonary hypertension, or any lung disease involving abnormal blood gases or requiring supplemental oxygen
    4. Renal insufficiency with an estimated glomerular filtration rate (eGFR) <30 ml/min/1.73 m^2^, or any history of renal dialysis or renal transplant
    5. Active malignancy or history of treated malignancy within 24 months of enrolment (other than cutaneous basal cell or squamous cell carcinoma)
    6. Clinically significant gastrointestinal problems involving the oesophagus or stomach including severe or erosive oesophagitis, uncontrolled gastric reflux, gastroparesis, oesophageal candidiasis or active gastroduodenal ulceration
    7. Active systemic infection
    8. COVID-19 disease
       1. Current confirmed, active COVID-19 disease
       2. Current positive test for SARS-CoV-2
       3. Confirmed COVID-19 disease not clinically resolved at least 3 months before the Consent Date.

Note: Participants with a prior asymptomatic infection and a negative test can be enrolled.

- 1. Other uncontrolled medical conditions that may modify device effect or increase risk, including uncontrolled diabetes mellitus (HgbA1c >8.0% if test result already obtained), untreated obstructive sleep apnoea or active alcohol abuse
  2. Predicted life expectancy less than one (1) year

1. Clinically significant psychological condition that in the Investigator’s opinion would prohibit the subject's ability to meet the protocol requirements/ Patient under legal protection
2. Current or anticipated enrolment in any other clinical study.
3. Employees/family members of:
   1. FARAPULSE or any of its affiliates or contractors
   2. The Investigator, sub-investigators, their medical office or practice, or healthcare organisations at which study procedures may be performed.
4. **Members of the** **BEAT-AF Study group**

**left during the study*

- **Trial Development Team (TDT):**

John Allison^1^, Besma Aouar^²^, Tiziri Aoudjit^2^, Julien Asselineau^2^, Laura Benkert^3^, Serge Boveda^4^, Christian Enzinger^5^, Hubert Cochet^6^, Isabel Deisenhofer^7^, Thomas Deneke^3^, Eric Frison^2^, Anne Gimbert^8^, Pierre Jaïs^6^, Josef Kautzner^9^, Sebastien Knecht^10^, Michelle Lycke^10^*, Philippe Maury^11^, Rozenn Mingam^6^*, Petr Neuzil^12^, Maider Piquet^6^, Sophie Regueme^8^*, Stephanie Roseng^6^, Marine Rousset^8^, Daniel Scherr^5^, Christopher Schneider^1^, Christine Schwimmer^2^, Maxime Sermesant^6^, Cedrick Wallet^2^, Dan Wichterle^9^

- **Clinical Trials Coordinating Team:**

Besma Aouar^2^, Tiziri Aoudjit^2^, Julien Asselineau^2^, Eric Frison^2^, Thomas Gil de muro^8^, Anne Gimbert^8^, Pierre Jaïs^6^, Maria Merched^2^, Laura Richert^2^, Marine Rousset^8^, Christine Schwimmer^2^, Cédrick Wallet^2^

Affiliations:

1 Boston Scientific, Menlo Park

2 Univ. Bordeaux, INSERM, Institut Bergonié, CHU de Bordeaux, CIC-EC 1401, Euclid/F CRIN Clinical Trials Platform, Bordeaux, France

3 RHÖN-KLINIKUM Campus Bad Neustadt

4 Clinique Pasteur, Toulouse

5 Medical University of Graz

6 IHU LIRYC, Univ. Bordeaux, CHU Bordeaux, France

7 Deutsches Herzzentrum München, Munich

8 CHU Bordeaux

9 Institute for Clinical and Experimental Medicine, Prague

10 AZ Sint-Jan Brugge-Oostende, Bruges

11 CHU Toulouse

12 Homolka Hospital, Prague

- **Trial Steering Committee (TSC):**

Pierre Jais, Bordeaux, France (Coordinating investigator – Chair)

Daniel Scherr, Graz, Austria (Austria representative, PI, Sub-study leader “Brain MRI”)

Sebastien Knecht, Bruges, Belgium (Belgium representative, PI, Sub-study leader “Redo procedures”)

Petr Neuzil, Prague, Czechia (Czechia representative, PI)

Thomas Deneke, Bad Neustadt, Germany (Germany representative, PI, Sub-study leader “Oesophageal safety”)

Anne Gimbert, Bordeaux, France (Sponsor representative - Head of “Internal Sponsor Department”)

Marine Rousset, Bordeaux, France (Sponsor Safety Officer)

Eric Frison, Bordeaux, France (Trial methodologist)

Julien Asselineau, Bordeaux, France (Trial statistician)

Hubert Cochet, Bordeaux, France (Sub-study leader “Atrial and ventricular function”)

Dan Wichterle, Prague, Czechia (Sub-study leader “Autonomic Nervous System”)

Trudie Lobban, Arrhythmia Alliance, UK (Patients representative)

John Morgan, Boston Scientific, USA (Boston Scientific representative)

Besma Aouar, Bordeaux, France (TSC Facilitator, EUCLID trial project manager)

Thomas Gil De Muro, Bordeaux, France (Sponsor - Regulatory representative)

Maxime Sermesant, Bordeaux, France (Complex Data coordinator)

Laura Richert, Bordeaux, France (EUCLID representative)

Christine Schwimmer, Bordeaux, France (EUCLID representative)

Cédrick Wallet, Bordeaux, France (EUCLID representative)

Andréa Alexander, Bordeaux, France (BEAT-AF Project Manager)

Christiane Andriamandroso*, Bordeaux, France (Liryc Grant Officer)

- **Data Safety and Monitoring Board (DSMB):**

Claire Duflos (Chair), CHU de Montpellier, Montpellier, France

Arnaud Denis, Clinique Saint Augustin, Bordeaux, France

Benoît Guy-Moyat, CHU de Limoges, Limoges, France

- **Site investigators (PI and co-PIs):**

**CHU Bordeaux**: Dr Nicolas Derval (PI), Pr Frederic Sacher, Dr Benjamin Bouyer, Dr Ghassen Cheniti*, Dr Meleze HOCINI, Pr Pierre Jaïs.

**CHU Toulouse**: Pr Philippe Maury (PI), Dr Anne Rollin.

**Clinique Pasteur, Toulouse**: Dr Serge Boveda (PI), Dr Stephane Combes, Jean-Paul Albenque.

**Institute for Clinical and Experimental Medicine, Prague**: Pr Josef Kautzner (PI), Dr Jana Haskova, Dr Petr Peichl, Dr Predrag Stojadinovic, Dr Dan Wichterle.

**Homolka Hospital, Prague**: Pr Petr Neuzil (PI), Dr Pavel Hala, Dr Jan PETRU.

**RHÖN-KLINIKUM Campus Bad Neustadt**: Pr Thomas Deneke (PI), Dr Elena Ene, Dr Karin Nentwich.

**Deutsches Herzzentrum München, Munich**: Pr Isabel Deisenhofer (PI), Dr Felix Bourier, Dr Florian Englert, Dr Nico Erhard, Dr Monika Hofmann, Dr Marc Kottmaier*, Dr Sarah Lengauer, Dr Tilko Reents, Dr Jan Syvari, Dr Marta Telishevska, Dr Alex Tunsch Martinez.

**Medical University of Graz**: Pr Daniel Scherr (PI), Dr Martin Benedikt, Dr Anna-Sophie Eberl, Dr Martin Manninger-Wuenscher, Dr Ursula Rohrer.

**AZ Sint-Jan Brugge-Oostende, Bruges**: Dr Sebastien Knecht (PI), Pr Mattias Duytschaever, Pr Jean-Benoît Le Polain de Waroux, Dr René Tavernier.

- **Sub-study leaders:**

Pr Thomas Deneke (Oesophageal Safety), Pr Daniel Scherr and Dr Christian Enzinger (Brain MRI Imaging), Dr Dan Wichterle (Autonomic Nervous System), Dr Sébastien Knecht (Redo procedures), Pr Hubert Cochet (Atrial and Ventricular Imaging).

- **Databasing and processing of complex data (IHU LIRYC, Univ. Bordeaux, CHU Bordeaux, France):**

Maxime Sermesant, Vigneshwar (Vicky) Gurunathan, Julien Castelneau.
